# Volatile emission in dry seeds as a way to probe chemical reactions during initial asymptomatic deterioration

**DOI:** 10.1093/jxb/erv568

**Published:** 2016-03-07

**Authors:** Sara Mira, Lisa M. Hill, M. Elena González-Benito, Miguel Angel Ibáñez, Christina Walters

**Affiliations:** ^1^Departamento de Biotecnología-Biología Vegetal, E.T.S.I. Agrónomos, Universidad Politécnica de Madrid, Madrid, Spain; ^2^USDA Agricultural Research Service, National Center for Genetic Resources Preservation, 1111 South Mason St, Fort Collins, CO 80524, USA; ^3^Departamento de Economía Agraria, Estadística y Gestión de Empresas, E.T.S.I. Agrónomos, Universidad Politécnica de Madrid, Madrid, Spain

**Keywords:** Fermentation, gas chromatography, methanol, pentane, peroxidation, seed aging, seed storage, seed quality markers, volatile organic compounds, water content.

## Abstract

The nature and kinetics of reactions in dry seeds determines how long the seeds survive. We used gas chromatography to assay volatile organic compounds (VOCs) emitted from seeds of three unrelated species as a means to non-invasively probe chemical changes during very dry, dry, and humid storage (seeds were dried to 5.5, 33, and 75% relative humidity at room temperature). VOCs emitted from seeds stored in humid conditions reflected fermentation-type reactions, with methanol and ethanol being predominant in *Lactuca sativa* and *Carum carvi*, and acetaldehyde and acetone being predominant in *Eruca vesicaria*. Dried *C. carvi* seeds continued to emit fermentation-type products, although at slower rates than the seeds stored in humid conditions. In contrast, drying caused a switch in VOC emission in *L. sativa* and *E. vesicaria* seeds towards higher emission of pentane and hexanal, molecules considered to be byproducts from the peroxidation of polyunsaturated fatty acids. Longevity correlated best with the rate of fermentation-type reactions and appeared unrelated to the rate of lipid peroxidation. Emission of VOCs decreased when seed species were mixed together, indicating that seeds adsorbed VOCs. Adsorption of VOCs did not appear to damage seeds, as longevity was not affected in seed mixtures. Collectively, the study shows similarity among species in the types of reactions that occur in dry seeds, but high diversity in the substrates, and hence the byproducts, of the reactions. Moreover, the study suggests that the most abundant VOCs arise from degradation of storage reserves within seed cells, and that these reactions and their byproducts are not, in themselves, damaging.

## Highlight

Species, storage products, and moisture have large effects on the nature and quantity of volatile emission from dry seeds, but storage time and seed viability do not.

## Introduction

Seeds are a major class of desiccation-tolerant organisms, which can survive in the absence of water. Metabolism changes dramatically in these organisms as water is removed, and there are few measurable reactions in cells containing less than 15% water (Walters *et al.*, 2005). Despite this highly restricted chemical activity, it is clear that some reactions occur in dry organisms, because they deteriorate with time and inevitably die. The effects of this deterioration, or aging, are usually identified as lost growth potential in rehydrated organisms, and the initial stage is extremely hard to detect. Eventually, a threshold is reached and very rapid deterioration ensues. In order to understand aging and predict when the threshold marking high morbidity will occur, we need a better understanding of the nature and kinetics of chemical activity in dry organisms.

Currently, several classes of chemical reactions are considered to occur under dry conditions, including fermentation, glycation (e.g. Maillard reaction), oxidation, and peroxidation, the latter reactions being initiated or stimulated by activated oxygen molecules or free radicals (Bailly, 2004; Colville *et al.* 2012; Job *et al.*, 2005; Kranner *et al.*, 2006, 2010; Labuza, 1980; Mira *et al.*, 2010; Walters *et al.*, 2010). Substrates for these reactions can be diverse, leading to a wide range of products that are classified into two main groups: (i) small molecular weight carbonyl compounds that escape into the airspace as volatile molecules and (ii) advanced glycation end-products, in which molecules become cross-linked (Chan, 1987; Frankel, 1983; Grosch, 1987; Halliwell and Gutteridge, 1999; Knutson *et al.*, 2000). These reactions usually occur at low rates in the dry state, and it remains unclear whether enzyme catalysis occurs. We speculate that the involvement of enzymes would narrow the types of substrates and products produced.

Because volatile organic compounds (VOCs) are a major byproduct of catabolic reactions, assessment of these molecules provides an accessible method to evaluate chemical reactions occurring in dry seeds. A growing number of studies have measured VOCs using gas chromatography (GC) or gas chromatography/mass spectrometry (GCMS) to monitor metabolism non-invasively. For example, volatile alka(e)nes and aldehydes are major byproducts of lipid peroxidation (Aldini *et al.*, 2010; Grotto et al, 2009; Rodríguez *et al.*, 1989). Pentane and ethane are commonly derived from oxidation of polyunsaturated fatty acids such as linoleic and linolenic acid, and have been used as biomarkers of aging in plant tissue culture (Rodríguez *et al.*, 1989), human breath (Mendis *et al.*, 1994), and human or animal tissues (Grotto *et al.* 2009; Halliwell and Chirico, 1993; Hartmut *et al.*, 1980). Saturated aldehydes, such as hexanal, are a potential biomarker of lipid peroxidation during storage of seeds (Colville *et al.*, 2012; Mira *et al.*, 2010; Zhang *et al.*, 1995a), animal tissues (Orhan *et al.*, 2006; Pignoli *et al.*, 2009), and human milk (Elisia and Kitts 2011). Lipid peroxidation reactions also yield unsaturated aldehydes such as hexenal and hydroxyalkenals (4-hydroxynonenal), which are common biomarkers of lipid peroxidation and oxidative stress (Aldini *et al.*, 2010; Grotto *et al.*, 2009).

Many VOCs are reactive and might be toxic, perpetuating reactions that lead to deterioration and accelerating the rate at which seeds lose viability, as has been confirmed for high concentrations of ethanol and acetaldehyde (Akimoto *et al.*, 2004; Zhang *et al.*, 1995a). Being potentially both a cause and an effect of deterioration, VOCs might serve as a biomarker for seed quality loss (Fielding and Goldsworthy, 1982; Hailstones and Smith, 1989; Smith and Adamson, 1989; Wilson and McDonald, 1986). Ethanol and methanol are reported to be major constituents of the airspace above seeds stored at a relative humidity (RH) of 65–90% (Akimoto *et al.*, 2004; Colville *et al.*, 2012; Lee *et al.*, 2000; Mira *et al.*, 2010; Obendorf *et al.*, 1990; Schwember and Bradford, 2005; Trawatha *et al.*, 1995; Zhang *et al.*, 1993, 1995a). In a previous study, we suggested that products reflecting glycolysis/fermentation-like reactions did not indicate specific damaging reactions, but rather indicated the fluidity of the glassy matrix in which aging reactions occurred (Mira *et al.*, 2010). In that work, we also showed a low correlation between the presence of lipid oxidation and peroxidation products and the rate of aging in lettuce seeds.

The present study continues to explore how measurement of VOCs might reveal details of chemical activity occurring in dry seeds. Our first objective was to explore ethanol and methanol emission in diverse species, to test whether these were ubiquitously produced and whether emission was regulated by water content during storage. We reasoned that a higher rate of VOC emission implies a higher reaction rate, which, in turn, would lead to faster deterioration. Hence, our second objective was to test the hypothesis that seeds with a poor shelf life emit more VOCs. We also wanted to test the hypothesis that physiologically relevant concentrations of volatile compounds were toxic and would promote faster aging. Without knowing the specific molecule to test, we opted to mix seeds, with the logic that a shorter-lived seed might emit compounds that would increase the rate of aging in a longer-lived seed. We used three species to make these comparisons, namely, *Lactuca sativa* (Asteraceae), *Eruca vesicaria* (Brassicaceae) and *Carum carvi* (Apiaceae).

## Materials and methods

### Seed material and measurement of lipid and water content

Lettuce seeds (*Lactuca sativa* L., cv. ‘Black-seeded Simpson’) were purchased from Gurney Seed Company, Greendale, IN, USA, in 2004 and 2009. Arugula seeds (*Eruca vesicaria* L. Cav.) were purchased from Richters in 2007. Caraway seeds (*Carum carvi* L.) were purchased from Hazzard’s Seeds in 2007. Seeds were stored at 5 °C and 30% RH until used for these experiments, which were initiated in 2007 for all the treatments (2800 days of storage). The experiment for *L. sativa* seeds (unmixed with other species) was repeated in 2009 using a 2009-harvested seed lot (2100 days of storage). All seed samples had high initial quality, with normal germination greater than 97%.

Total lipid content was measured using a protocol modified from Bligh and Dyer (1959). Pre-weighed samples of ground seeds (0.5–2.0g) were mixed in a chloroform:methanol (2:1) solution for 10min. The solvent was collected and the pellet rewashed in chloroform:methanol solution two more times. After separation, the bottom layer (i.e. the solvent and dissolved lipids) was retained and washed twice with 1:1 methanol:0.9% NaCl solution. The solvent fraction was then evaporated to remove chloroform. The remaining lipids were weighed and the amount of lipid was calculated per gram of seed dry weight (dw). Values are expressed as the average of two replicate extractions. Fatty acids were esterified using the protocol of Metcalfe and Schmitz (1961) and then characterized using GC. The fatty acid derivatives were separated on a gas chromatograph with a flame ionization detector (FID; model 8500, Perkin-Elmer, Waltham, MA, USA) using a Supelco Nukol 30 m, 0.25mm internal diameter fused silica capillary column (Sigma-Aldrich, St. Louis, MO, USA). Helium was used as the carrier gas at flow rates set to 0.14MPa (20 psi). Injector and detector temperatures were set to 220 °C and oven temperature was 100 °C increasing to 190 °C at 10 °C min^−1^.

### Seed storage

Experiments consisted of adjusting the water content of seeds, hermetically sealing seeds in vials, storing vials at 35 °C, and sampling intermittently for airspace analysis, seed viability, and seed water content. Seed water content was adjusted by maintaining seeds at different RH at 25 °C. RH was controlled in desiccators containing saturated solutions of ZnCl_2_ (very dry: 5.5% RH), MgCl_2_ (dry: 33% RH) or NaCl (humid: 75% RH) (Vertucci and Roos, 1993). Water content was determined from a comparison of sample mass (sample size ranged from 0.01–0.09g) before and after drying at 95 °C for 72h using a microbalance (Orion Cahn C-33, Thermo Fisher Scientific, Inc., Beverly, MA, USA) and is expressed as g H_2_O g^−1^ dw. Water content at the beginning of the experiment was measured on two replicated samples prior to sealing vials and then periodically during the storage experiment as a test that vials were properly sealed. The three RH treatments gave water content ranges of 0.030–0.039, 0.042–0.063, and 0.089–0.131g H_2_O g^−1^ dw.

Approximately 30 aliquots of 50 seeds each (0.15g for *L. sativa* and 0.6g for *E. vesicaria* or *C. carvi*) were sealed into individual 2.7ml crimp-top vials (National Scientific, Rockwood, TN, USA). Seeds of *L. sativa* (0.15g) were also mixed with seeds of *E. vesicaria* or *C. carvi* (0.6g) and sealed into crimp-top vials. All vials were placed at 35 °C and samples were removed for testing periodically over a 7.5-year (2800 days) period.

### Germination tests and seed longevity

Deterioration of seeds during storage was detected by changes in percentage germination. Seeds from a stored vial were sown on 14cm Petri plates containing 1% agar and incubated under a 16h light/8h dark cycle at 20 °C for 7 days for *L. sativa* and *E. vesicaria* seeds, and at 25 °C for 14 days for *C. carvi* seeds. Petri dishes were photographed after the incubation period and germination was measured using ImageJ software (Rasband, 1997–2008). Each germination assay consisted of two replicates of 25 seeds. In the first series of experiments in which longevity among species was compared, the 2009-harvested *L. sativa* seed lot was used, and this gave us comparisons of seeds tested within 6 months of harvest. The second series of experiments was initiated in 2007 and the 2004-harvested *L. sativa* seed lot was mixed with 2007-harvested *E. vesicaria* or *C. carvi* seeds. Germination time-course data for the unmixed 2004-harvested cohort of *L. sativa* seeds were presented previously (Mira *et al.*, 2010), with sampling times for the dry treatment added.

The response to storage time in terms of percentage germination was modeled using the glm function with a binomial distribution available in the statistical package R (R Core Team, 2015). Time for seed viability to decline (i.e. longevity) was determined for each species, humidity treatment, and replicate. Time for germination percentage to decrease to 75% or 50% of maximum (P75 and P50, respectively) was calculated using the dose.p function available in R (R Core Team, 2015). We used the value of P75 to indicate the duration of the initial asymptomatic phase of seed deterioration.

### Characterization of volatile production from seeds

Volatile compounds emitted by seeds during storage were characterized using GC. Periodically, a crimp-top vial containing seeds was removed from storage at 35 °C and allowed to cool to room temperature for 1–2 hours before a 1ml sample of air was taken from the headspace of the vial using a gas-tight syringe (Hamilton, Reno, NV, USA). The air sample was injected directly into the gas chromatograph (Perkin Elmer, Autosystem XL, Norwalk, CT, USA). Injector and FID detector temperatures were set at 200 °C; helium was used as the carrier gas and set at a flow rate of 0.8ml min^−1^. Volatile compounds were separated using a 30 m, 0.32mm internal diameter DB624 capillary column (Agilent Technologies, Santa Clara, CA, USA) that was held at 35 °C for 5 minutes, increased at 8 °C min^−1^ to 200 °C, and then held at 200 °C for 10min. Volatile compounds were identified by retention times (RTs) of known standards. We measured RT for 58 standards, 33 of which were also included in the manufacturer’s list of RTs for 216 non-halogenated compounds (Agilent Technical Overview 5991-5017EN, 2014). RTs for all analytes were time-corrected by pentane and/or acetone (RT = 6.05 and 7.26, respectively), which were reliably detected in most samples, obviating the need for an internal standard.

To confirm compound identity, a subset of eight samples was also analyzed using GCMS by an outside analytical laboratory (Edison Analytical Laboratories Inc., Schenectady, NY, USA). Vials representing different species and humidities that had been stored for 2–3 years were shipped to the laboratory in an insulated box to prevent major temperature changes. Volatile compounds were collected on to a solid-phase microextraction fiber (50/30 μm DVB/Carboxen/PDMS; Supelco, Bellefonte, PA, USA) that was inserted through the septum of the sample vial and held in place with a support for 40min at room temperature. The fiber was then desorbed for 2min in the injection port set at 260 °C of a HP5890 GC connected to a HP5972 mass selective detector (Hewlett Packard/Agilent, Santa Clara, CA, USA). Separation by GC was conducted using a DB624 capillary column (Agilent Technologies) held at 35 °C for 5min, increased at 8 °C min^–1^ to 200 °C, and then held at 200 °C for 10min. Library searches of analytes used the Wiley/NIST library of mass spectra (2008). A total of 56 compounds were identified by GCMS, of which 32 were included as VOC standards as described above.

A library of 266 compounds and associated RTs specific to our GC protocols was constructed using the 58 standards we tested and translating the 184 RTs for compounds unique to the manufacturer and 24 compounds unique to the GCMS analyses with correlation models of the ~30 standards we had in common with the manufacturer’s list and GCMS data. In addition, we developed relationships between RTs and carbon length for different carbonyl groups (alcohols, alkanes, aldehydes, acids, esters, diols, diones, and di-oxy and -ene groups) and used these relationships to infer RTs of compounds not included in any list.

Peaks with area greater than 225 µVs were analyzed. This high sensitivity allowed us to detect compounds emitted in very low quantities. Previously published data for *L. sativa* seeds containing 0.089g g^−1^ water content (Mira *et al.*, 2010) were reanalyzed using this more sensitive threshold. VOC levels were calculated from peak size and relationships established between peak size and carbon chain length for different carbonyl groups (Mira *et al.*, 2010). Kinetic models of volatile production were developed by linear regression of moles VOC emitted and storage time.

## Results

Lipid composition varied among seed species. Seeds of *L. sativa* had the highest total lipid content (32%) and seeds of *C. carvi* the lowest (10%) (Table 1). Linoleic and oleic acids were prominent in seeds of all three species. Seeds of *E. vesicaria* also had high levels of erucic acid (Table 1). At any specific RH, water content was higher in seed species that had a lower lipid content (Table 2).

**Table 1.** Percentage of fatty acid in total lipids of seeds of three species

Fatty acids (%±SE)	Chain length	*L. sativa* (2004)	*L. sativa* (2009)	*E. vesicaria*	*C. carvi*
Lauric	C12	0	0	0	2.9±0.4
Myristic	C14	0.2±0.1	0	2.3±1.0	0
Palmitic	C16	9.9±0.6	8.8	7.3±0.9	6.1±0.7
Palmitoleic	C16:1	0	0	0	0
Stearic	C18	3.2±0.1	3.2	1.3±0.1	1.2±0
Oleic	C18:1	25.5±0.2	26.7	18.1±1.1	52.5±4.3
Linoleic	C18:2	58.6±0.5	60.3	12.2±1.4	32.7±1.0
Linolenic	C18:3	0.2±0.1	0	13.2±0.1	0.3±0.1
Arachidate	C20	0.8±0.1	0.4	0.7±0.0	4.2±4.1
Eicosenoate	C20:2	0	0	9.3±2.6	0
Behenic	C22	1.6±0.9	0.6	1.3±1.3	0
Erucic	C22:1	0	0	34.4±3.2	0
Lipid content		32±6	33±0.3	19±0	10±0

**Fig. 1.**
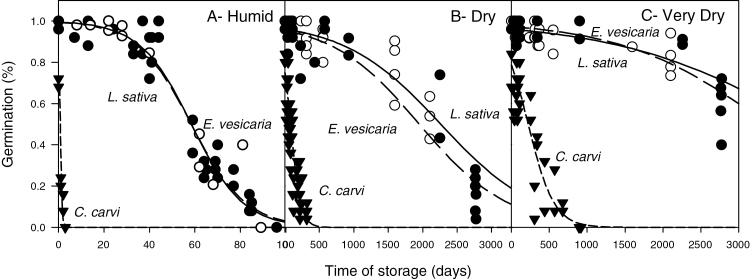
Changes in percentage germination of *L. sativa* (solid curve), *E. vesicaria* (long dash), and *C. carvi* (short dash) seeds during storage at 35 °C and three moisture treatments: (A) Humid (0.099–0.131g g^–1^), (B) Dry (0.042–0.063g g^–1^), and (C) Very Dry (0.030–0.039g g^–1^). Each point represents a germination assay in which the percentage of normal seedlings was measured for a particular treatment, storage time, and replicate. Data were fitted to a logistic regression model and values for 75 and 50% seed germination (P75 and P50) were calculated (Table 2).

**Table 2.** Kinetics of deterioration in seeds stored at 35 °C and different water contents. Deterioration is expressed as the time for seed quality to decrease to 75 and 50% of initial germination (P75 and P50, respectively)

Treatment	Species	Water content (g g^-1^ dw±SE)	Seed longevity (days±SE)	
			P75	P50
Humid	*L. sativa*	0.099±0.003	46±1	58±1
	*E. vesicaria*	0.100±0.001	45±1	58±1
	*C. carvi*	0.131±0.001	0.4±0.1	0.8±0.1
Dry	*L. sativa*	0.042±0.001	1 486±73	2 261±113
	*E. vesicaria*	0.048±0.001	1 244±77	1 952±80
	*C. carvi*	0.063±0.001	1±8	81±4
Very dry	*L. sativa*	0.030±0.001	2 537±3.805	3 882±518
	*E. vesicaria*	0.034±0.001	2 334±125	3 379±195
	*C. carvi*	0.039±0.001	35±17	222±14

Changes in germination with storage time followed the characteristic reverse-sigmoidal time course, which starts with an initial asymptomatic stage and concludes with rapid loss in seed viability (Fig. 1). Water content affected the duration of the asymptomatic phase. At 35 °C, seeds stored under humid conditions lost capacity to germinate within 3–100 days, depending on species (Fig. 1A). In contrast, seeds stored under dry and very dry conditions maintained high germination percentages for ~2000 (*L. sativa* and *E. vesicaria*) and 50 (*C. carvi*) days (Fig. 1B, C).

The initial asymptomatic stage of seed deterioration was considered to be the time before significant loss in viability and characterized as the time for germination to decrease to 75% of maximum germination (P75). Duration of P75 ranged from 1 to 2545 days depending on water content during storage and species (Table 2). P75 increased with decreasing water content for all three seed species (*P*≤0.0005). Within each water content treatment, *C. carvi* aged significantly faster than *L. sativa* and *E. vesicaria* (*P*≤0.0005). The latter two species had similar longevities under all storage conditions (Fig. 1; Table 2).

**Fig. 2.**
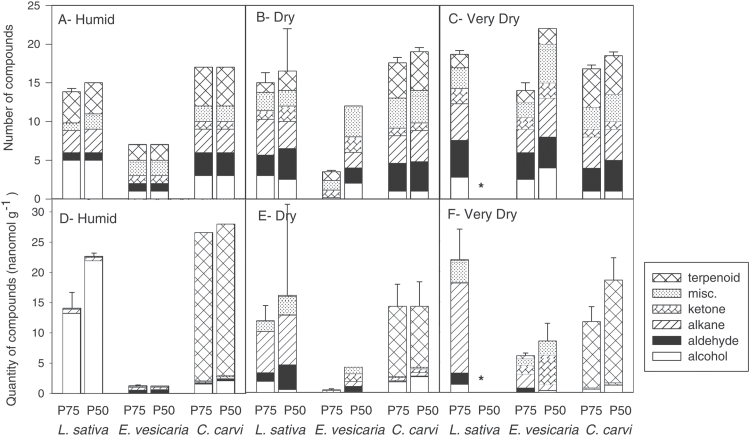
Volatile emissions by *L. sativa*, *E. vesicaria*, and *C. carvi* seeds during storage at 35 °C and three moisture treatments: (A, D) Humid (0.099–0.131g g^–1^), (B, E) Dry (0.042–0.063g g^–1^), and (C, F) Very Dry (0.030–0.039g g^–1^). The number and quantity of VOCs are given as seed germination decreased to 75 and 50% (P75 and P50). The number of molecular species (A–C) and total molar quantity emitted (D–F) were classified by the carbonyl group. Data were not available for the treatment indicated by asterisks because samples were depleted before sufficient deterioration was detected. misc, Miscellaneous.

Different compounds, varying in carbon chain length and carbonyl group, were detected in the airspace above stored seeds and identified as 1–10 C acid, alcohols, aldehydes, alkanes, furans, ketones, and terpenes. To facilitate comparisons at similar stages of deterioration, VOC composition was evaluated by averaging samples with germination between 100 and 75% (P75) and samples with germination between 75 and 50% (P50) (Fig. 2). Depending on species and water content, between seven and 23 compounds were detected in the vial airspace. *L. sativa* and *C. carvi* emitted a similar number of compounds for each water content treatment (~15–20), while the number of compounds emitted from *E. vesicaria* varied from 4 to 24 depending on the humidity and extent of deterioration (Fig. 2A–C).

**Fig. 3.**
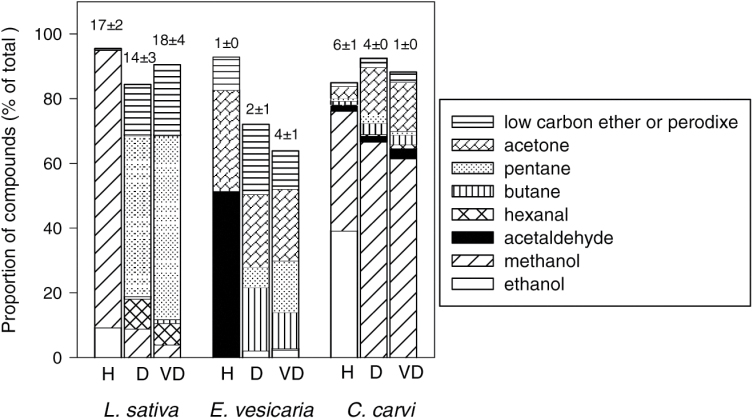
Volatile emissions by *L. sativa*, *E. vesicaria*, and *C. carvi* seeds during storage at 35 °C and three moisture treatments: Humid (H, 0.099–0.131g g^–1^), Dry (D, 0.042–0.063g g^–1^), and Very Dry (VD, 0.030–0.039g g^–1^). Bars represent average quantities (in percentage over total quantity) among storage times (<1200 days or when monitoring stopped) of major components: ethanol, methanol, acetaldehyde, hexanal, butane, pentane, acetone and a low-carbon ether or peroxide. Values above the bars are the average of total quantity of VOCs emitted (nmol g^−1^). Total VOCs included all detected compounds except terpenes; bars do not sum to 100% because amounts of individual minor compounds are not shown.

The total amount of emitted VOCs ranged among species and water contents (Fig. 2D–F, also summarized by the numbers shown above the bars in Fig. 3). The VOC concentration at P75 and P50 for humid storage was greatest for *C. carvi* (~27 nmol g^−1^ seed) and least for *E. vesicaria* (~1 nmol g^−1^ seed). For drier storage, VOC concentration at P75 and P50 was lower for *C. carvi,* comparable to *L. sativa* (~15 nmol g^−1^ seed), and higher for *E. vesicaria* seeds (~5 nmol g^−1^ seed) compared with counterparts stored under humid conditions. Data for VOC emission at P50 for *L. sativa* seeds stored under very dry conditions are not available because all samples were used before P50 was reached (i.e. >2100 days). Volatile composition in the humid-stored seeds at P25 and when all seeds had died appeared similar to our observations at P75 and P50 (data not shown), confirming our previous report (Mira *et al.*, 2010). The error bars in Fig. 2 represent variation among vials sampled before P75, and between P75 and P50. While the number of VOCs detected appeared consistent among samples within a given time period (Fig. 2A–C), the quantity of VOCs varied greatly among samples, especially for the dry and very dry treatments (Fig. 2E, F), which were necessarily longer storage times.

In order to confirm the consistent prominence of certain analytes, despite variation in the total amount (Fig. 2E, F), we analyzed GC profiles by the quantity of compound (nmol g^–1^) relative to the total quantity of compounds in the sample. For this analysis, we pooled data from samples that had been stored for ≤1200 days (*L sativa* and *E. vesicaria*) or upon completion of the germination time course (*C. carvi*), a treatment that we felt was justified because there were no perceived time-trends in VOC composition during this period (data not shown). The types of compounds emitted from seeds varied among species and moisture treatments. A few compounds were added to the list already published for lettuce (Mira *et al.*, 2010) as a result of a higher sensitivity threshold. Alcohols were the major constituent in the airspace above humid-stored *L. sativa* seeds (Fig. 2D, water content 0.099g g^−1^), with ethanol and methanol comprising ~90% of detected molecules (Fig. 3). The VOC profile of dry-stored *L. sativa* seeds was markedly different from that of those exposed to the humid treatment, with low alcohol content, high aldehyde and alkane content, and several minor molecules (water content ≤0.042g g^−1^, Fig. 2E, F; see also Fig. 3). Pentane, hexanal, and an as yet unidentified molecule with low RT, believed to be a short-chain ether or peroxide, were consistently detected in dry *L. sativa* samples. The headspace composition of vials containing *E. vesicaria* seeds differed substantially from profiles from *L. sativa* seeds (Fig. 3), with acetaldehyde and acetone comprising over 83% of detected VOCs in the humid treatment. Acetaldehyde was replaced by butane and pentane in VOCs above dry *E. vesicaria* (Fig. 3), and compounds such as butanal or butanone appeared and were more pronounced than was observed from *L. sativa*. Most of the compounds emitted by *C. carvi* were terpenes and terpenoids (e.g. carvone, limonene, α-pinene, β-pinene) (Fig. 2D–F), which are interpreted as flavor compounds rather than degradation products *per se* (Tammela *et al.*, 2003). To facilitate observations of molecules believed to be associated with changes in seed quality, the concentrations of terpenes were subtracted from the total VOCs measured and are omitted from further analyses. Once this correction was made, it was clear that, as for *L. sativa* seeds, methanol and ethanol were prevalent compounds in the headspace above humid-stored *C. carvi* seeds, and, unlike *L. sativa* seeds, methanol comprised a large proportion of VOC emission in dry-stored *C. carvi* seeds (Fig. 3). During dry storage, this species also emitted butane, pentane, acetone, and the unidentified short-chain ether or peroxide that was also observed for *E. vesicaria* and dry *L. sativa* seeds. For all three species, ~10% of total VOCs from dry-stored seeds was composed of several minor compounds.

**Fig. 4.**
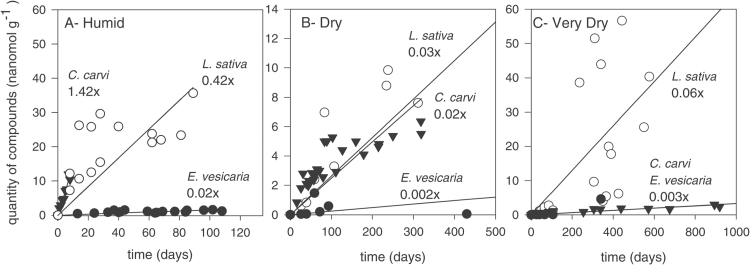
Time course for total production of volatile compounds (except terpenes) emitted by *L. sativa*, *E. vesicaria*, and *C. carvi* seeds stored at 35 °C and three moisture treatments: (A) Humid (0.099–0.131g g^–1^), (B) Dry (0.042–0.063g g^–1^), and (C) Very Dry (0.030–0.039g g^–1^). Values correspond to the slope for each species and treatment.

Initial accumulation of total VOCs (except terpenes) was characterized for each seed species and moisture environment (Fig. 4). Under humid conditions, VOC levels increased linearly with storage time, and the slope for *C. carvi* seeds was greater than those for the other two species (slopes were 1.42, 0.42, and 0.02 nmol g^−1^ day^−1^ for *C. carvi, L. sativa*, and *E. vesicaria* seeds, respectively) (Fig. 4A). VOC emission continued unabated even after all seeds had died. For the dry treatment, VOC emission increased with storage time for *L. sativa* and *E. vesicaria* seeds throughout the storage period, although data are fairly scattered (Fig. 4B; data for *L. sativa* and *E. vesicaria* seeds at storage time >500 days are not shown). VOC emission appeared to level off in vials containing *C. carvi* seeds between 100 and 320 days, when monitoring ended (Fig. 4B). Calculations of slopes suggested comparable emission rates for *C. carvi* and *L. sativa* (0.02 and 0.03 nmol g^−1^ day^−1^, respectively) and considerably slower emission for *E. vesicaria* seeds (0.002 nmol g^−1^ day^−1^; Fig. 4B). For the very dry treatment, VOC emission increased with storage time (Fig. 4C; data for all species not shown at storage time >1000 days). The emission rate for very dry *C. carvi* seeds was much reduced compared with emission rates under other moisture treatments (slope was 0.003 nmol g^−1^ day^−1^) and was comparable to emission rates for *E. vesicaria* seeds at this moisture level (slope 0.003 nmol g^−1^ day^−1^). VOC data for very dry *L. sativa* were highly scattered, but suggested faster emission in very dry compared with dry storage (slope was 0.06 nmol g^−1^ day^−1^), contrasting with observations made for very dry *C. carvi* or *E. vesicaria* seeds.

**Fig. 5.**
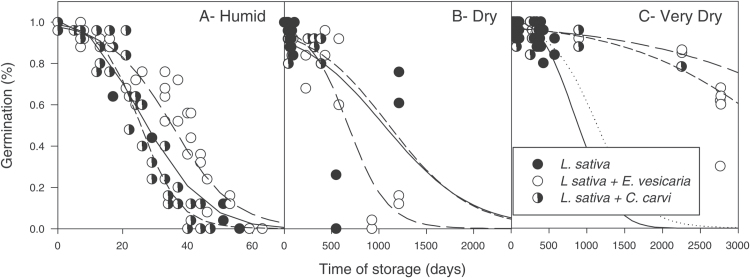
Changes in percentage germination of *L. sativa* when stored alone (solid curve) and in the presence of *E. vesicaria* (long dash) or *C. carvi* (short dash) seeds at 35 °C and three moisture treatments: (A) Humid (0.090–0.086g g^–1^); (B) Dry (0.048–0.042g g^–1^), and (C) Very Dry (0.033–0.029g g^–1^). Data were fitted to a logistic regression model and values for P75 and P50 were calculated (Table 3). The dotted line in (C) corresponds to a different extrapolation of longevity for the same dataset. Water contents are for *L. sativa* seeds only and there is slight variation between seeds alone and in mixtures.

To test the hypothesis that volatile production from seeds affected longevity, we compared viability within seed mixtures of *L. sativa* and either *E. vesicaria* or *C. carvi*. The longevity of seeds was not significantly affected by the presence of another species in most cases (Fig. 5; also compare P75 values in Table 3 and those for *E. vesicaria* and *C. carvi* in Table 2). Results for *L. sativa* seeds in mixtures with *E. vesicaria* were inconsistent, showing slightly beneficial effects of mixing under humid conditions (*P*≤0.0005), slightly detrimental effects under dry conditions, and beneficial effects under very dry conditions (Fig. 5, Table 3). There is high uncertainty in the longevity values for *L. sativa* alone (very dry treatment, Fig. 5C) and in combination with *C. carvi* (dry treatment, Fig. 5B), because these were extrapolated from sampling times <1000 days.

**Table 3.** Kinetics of deterioration in seed samples of pure and mixed mixtures stored at 35 °C and different water contents. Deterioration is expressed as the time for seed quality to decrease to 75 and 50% of initial germination (P75 and P50, respectively). Time course data for *L. sativa* seeds (2004 cohort) is provided in Fig. 5. Data for unmixed *L. sativa* seeds are from Mira *et al.* (2010) with additional sample times for the dry treatment added

Treatment	Species/mixture	Water content (g g^–1^ dw±SE)	Seed longevity (days±SE)	
			P75	P50
	*L. sativa*	0.089	16±4	27±4
	*L. sativa* (*+E. vesicaria*)	0.086	25±1	35±1
	*L. sativa* (*+C. carvi*)	0.090	19±1	25±1
	*E. vesicaria* (+*L. sativa*)	0.102	42±1	55±1
	*C. carvi* (*+L. sativa*)	0.125	0.3±0.1	0.9±0.1
Dry	*L. sativa*	0.046	550±67	1 056±109
	*L. sativa* (*+E. vesicaria*)	0.046	445±29	675±34
	*L. sativa* (*+C. carvi*)	0.048	643±175	1 108±331
	*E. vesicaria* (+*L. sativa*)	0.048	1 178±66	1 703±73
	*C. carvi* (*+L. sativa*)	0.064	7±6	76±4
Very dry	*L. sativa*	0.030	567±70	882±18^a^
	*L. sativa* (*+E. vesicaria*)	0.029	3 021±753	4 470±1 185
	*L. sativa* (*+C. carvi*)	0.033	2 341±475	3 407±723
	*E. vesicaria* (+*L. sativa*)	0.036	2 281±132	3 379±203
	*C. carvi* (*+L. sativa*)	0.043	61±17	225±17

^a^ This value was obtained by extrapolating a logistic model beyond the last sampling time of 550 days (Mira *et al.*, 2010).

**Fig. 6.**
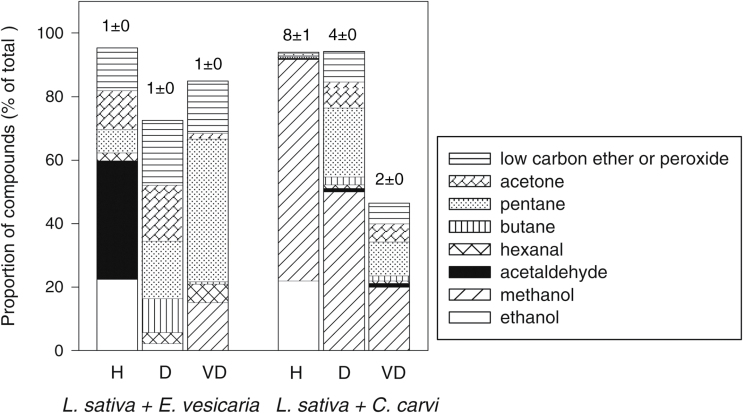
Volatile emissions from seed mixtures containing *L. sativa*+*E. vesicaria* and *L. sativa*+*C. carvi* seeds stored together at 35 °C and three moisture treatments as described in Fig. 5. Bars represent the average quantities of major components of the volatile profile: ethanol, methanol, acetaldehyde, hexanal, butane, pentane, acetone and a low-carbon ether or peroxide. Values above the bars are total VOC levels (nmol g^−1^) averaged for storage times less than 1200 days. Total VOCs included all detected compounds except terpenes; bars do not sum to 100% because amounts of individual minor compounds are not shown.

Although longevity in seed mixtures appeared mostly unaffected, the composition of compounds in the headspace was different in vials containing a combination of seed species compared with vials containing a single species. The total amount of compounds detected in the headspace when *L. sativa* seeds were mixed was not additive as we expected, but rather was characteristic of *E. vesicaria* or *C. carvi* seeds alone (most treatments) or intermediate between *L. sativa* and *C. carvi* (humid treatment; compare numbers above bars in Fig. 3 and Fig. 6). VOC composition reflected a mixture of prevalent molecules from both species averaged in the airspace of the mixture (Fig. 6). Two exceptions to this observation are noted: (i) a complete loss of the methanol signal (from *L. sativa*) in the humid and dry treatments of *L. sativa* mixed with *E. vesicaria* seeds, and (ii) an increase in minor compounds in the very dry *L. sativa*+*C. carvi* mixture (Fig. 6). Another notable difference in VOC composition in *L. sativa*+*C. carvi* mixtures was the diminished presence of terpenes, which were highly abundant in *C. carvi*-only samples (data not shown).

## Discussion

In this paper, we address the problem of characterizing the nature and kinetics of reactions that occur in dry biological systems. The problem is difficult because reactions tend to be slow (detection time is days to years) and because subtle chemical changes are difficult to discern through small changes in substrates. Further, dry biological systems are amorphous solids in which the reaction rate is typically regulated by both the concentration of substrate and structural relaxation or localized organization of substrates or reaction sites. This interaction confounds direct correlations between kinetics and substrate levels or the presence of catalysts (Walters *et al.*, 2010). Characterizing the nature and kinetics of reactions in dry systems is important in order to understand slow changes in physiology in organisms that are typically regarded as inert. The practical goal of this research is to gain better insight into the mechanisms by which seeds deteriorate and eventually die during dry storage.

To focus on reaction products, rather than substrates, and to exploit the rare opportunity of using water as an experimental variable rather than a component of the analysis, we explored the production of VOCs from seeds. Analysis of VOCs in breathalyzer tests for animals is increasingly being used to identify reactions associated with aging or pathology (Aldini *et al.*, 2010; Grotto *et al.*, 2009; Hartmut *et al.*, 1980). VOC analysis accommodates small sample size and does not require destructive sampling of tissues. In a previous study using *L. sativa* seeds, we established prominent VOCs produced by these seeds under humid and dry conditions and demonstrated that drying to ~30% RH induces a switch away from fermentation-type reactions and towards peroxidation-type reactions (Mira *et al.*, 2010). We also suggested that lower rates of VOC production in seeds dried to 30% RH reflected the loss of molecular mobility in solidifying cellular matrices. Questions arising from that study include the apparent increase in peroxidation products as seeds are progressively dried below 30% RH and the poor correlation between peroxidation products and rate of aging, despite current wisdom that aging is a consequence of oxidation (Bailly, 2004; Groot *et al.*, 2015; Job *et al.*, 2005; Kranner *et al.*, 2006; McDonald, 1999; Walters, 1998; Walters *et al.*, 2010). In addition, other laboratories studying VOCs emitted from seeds (Colville *et al.*, 2012; Zhang *et al.*, 1993, 1995a) have indicated a broader array of molecules than we previously detected. Our goal in the present study was to address some of these questions by using additional species and greater sensitivity in our GC methods.

The VOC analysis technique is fraught with problems of sensitivity and interpretation. Dynamic interactions among compounds emitted into the headspace influence the interpretation of the nature and kinetics of reactions occurring in the solid matrix below (Aldini *et al.*, 2010; Grotto *et al.*, 2009; Halliwell and Chirico, 1993). For example, ethanol and acetaldehyde may interconvert through alcohol dehydrogenase, and pentane might convert to pentanol. Molecules may be highly unstable and change rapidly, leading to multiple or undetected signals. Some byproducts of lipid peroxidation, such as malondialdehyde and 4-hydroxynonenal, are so unstable that derivatization to a more stable compound is required for detection (Aldini *et al.*, 2010; Grotto *et al.*, 2009; Halliwell and Chirico, 1993; Zhang *et al.*, 1993). In addition, it is difficult to give unambiguous assignments of isomers with similar RTs, which occurs more frequently as RT and molecular mass increase (Mendis *et al.*, 1994). Very small molecules (e.g. alkanes and ethers with three or fewer carbon atoms) were not detectable using our protocols because they elute in the void volume.

Quantification of VOCs produced by chemical reactions in seeds is confounded by adsorption–desorption relationships between VOCs and molecular interfaces. Cells naturally contain compounds and structures analogous to solid-phase microextraction fibers that trap VOCs, and we should expect these interactions to influence VOC quantification. Early work has considered the kinetics of VOC production and sorption/desorption in animal (Harmut *et al.*, 1980) and seed (Zhang *et al.*, 1995a) cells, but more work is needed to accurately model the partitioning of VOC products between solids and airspaces of storage containers. In this study, we demonstrate that some seeds, such as *E. vesicaria* or *C. carvi,* can adsorb VOCs produced by *L. sativa* seeds and effectively lower the VOC concentrations in the airspace (compare the total emission values in Fig. 6 with those in Fig. 3). From this experiment, we conclude that accurate quantification of VOC production in seeds must include considerations of sorption–desorption dynamics. The seminal literature in this regard comes from studies of seed fumigation (Lubatti and Smith, 1948). In general, our study supports work showing a relationship between storage atmosphere and changes in physiology (Gonzalez-Benito *et al.*, 2011; Groot *et al.*, 2015; Halliwell and Chirico, 1993), but there is a need for more in-depth analyses to establish the bases for these relationships.

VOC analyses reflect the most prominent types of reactions, but these may not be the most physiologically relevant. For example, the high production of pentane in *L. sativa* seeds (Fig. 3) indicates peroxidation of linoleic acid (Frankel, 1983; Knutson *et al.*, 2000). High rates of this reaction probably reflect the high level of available substrate in the form of storage reserves in the seed (Table 1) rather than a reaction with pathological consequences. That said, pentane production might reflect the fluidity of lipid bodies and, if so, suggests an interesting probe of the non-aqueous environment within seeds and water interactions. Future experiments will monitor the kinetics of alkane production in seeds with varying lipid content and liposome structure and in response to temperature changes, with the goal of describing reaction kinetics as non-aqueous cellular components solidify. It may be that the minor constituents of the GC profile more directly describe aging or pathological reactions in seeds. Degradation of substrates in lower abundance than storage reserves would appear as minor compounds. These are more difficult to characterize because they are barely detectable and appear inconsistently among chromatograms. Previously, we overlooked many of these molecules as noise in the chromatogram baseline. Indeed, baselines become increasingly ‘messy’ as storage time progresses (data not shown).

Previously, we reported high production of ethanol and methanol in *L. sativa* seeds, diminishing emission rates as seed moisture decreased from 0.09 to 0.05g H_2_O g^−1^ dw, and a strong correlation between emission rate and aging rate within this range of moisture content (Mira *et al.*, 2010). We postulated that these molecules arose from fermentation-type reactions and that emission kinetics reflected decreasing molecular mobility within drying seeds. In the present study, we investigated whether these fermentation products are ubiquitously produced in seeds in the humid to dry moisture range and whether rate of emission corresponds with aging rate. Methanol and ethanol production was also observed in *C. carvi* seeds (Fig. 3) and has previously been reported in other species (Bicanic *et al.*, 2003; Buckley and Buckley, 2009; Colville *et al.*, 2012; Kodde *et al.*, 2012; Lee *et al.*, 2000; Min, 2012; Mira *et al.*, 2010; Rutzke *et al.*, 2008; Taylor *et al.*, 1999; Zhang *et al.*, 1993, 1994, 1995b; Zhang and Roos, 1997). In contrast, seeds of *E. vesicaria* produced acetaldehyde and acetone under humid conditions (Fig. 3), molecules that have been linked to fermentation of pyruvate in single-cell organisms, especially in low-oxygen and high-metal contexts (Jardine *et al.*, 2010; Jensen, 1976; Lehninger *et al.*, 1993). Differences in fermentation products (if these reactions are, indeed, related to fermentation) among species suggest different substrates or modes of catalysis; both possibilities require further investigation to gain insights about the nature of reactions in drying seeds. Emission of the supposed fermentation products was fastest for *C. carvi* under humid conditions (Fig. 4A), and this species was also the fastest to age among our study samples (Fig. 1A); however, *L. sativa* and *E. vesicaria* aged at similar rates and had considerably different emission rates.

The presence of ethanol and methanol in the headspace above stored seeds has been implicated as a marker of seed quality for some species (Buckley and Buckley, 2009; Kodde *et al.*, 2012; Rutzke *et al.*, 2008; Taylor *et al.*, 1999; Zhang *et al.*, 1993, 1994, 1995b; Zhang and Roos, 1997). However, we do not believe these molecules are a direct cause of poor quality. *E. vesicaria* adsorbed VOCs produced by *L. sativa*, and there was no detrimental effect on longevity (Tables 2 and 3). The slight improvement in longevity of *L. sativa* seeds when mixed with *E. vesicaria* seeds (Fig. 5A) may have arisen from *E. vesicaria* scavenging these molecules or from the slight drying that was also indicated.

The distinct change in the VOC profile when *L. sativa* and *E. vesicaria* seeds were dried to <0.065g g^−1^ water content (the dry and very dry treatments; Figs 2 and 3) suggest a switch in the types of reactions that occur. We suggest this arises from reactions in aqueous domains of seed cells becoming increasingly restricted and reactions in lipid domains becoming increasingly facilitated as seeds dry. The continued emission of methanol from dry and very dry *C. carvi* seeds suggests a different response to drying, and this might contribute to the overall poor longevity of seeds of this species.

We present longevity data for two cohorts of *L. sativa* (cv. ‘Black Seeded Simpson’) seeds that differ substantially (Tables 2 and 3) even though storage conditions were similar. We suggest that the primary reasons for these differences are that (i) the 2004 cohort was stored for 3 years before it was used, while the 2009 cohort was used within 6 months of harvest, and (ii) there is uncertainty in longevity predictions when storage time is considerably less than response time. Extending storage time data for the dry treatment (Table 3) increased P75 and P50 estimates compared with those reported by Mira *et al.* (2010), and we would expect to observe a similar effect on the longevity estimates of the very dry treatment if data for extended times were available. Other minor effects include variation in storage condition, maternal effects, post-harvest handling differences, and intrinsic variation in longevity values as longevity increases.

## Conclusions

Emission of volatile compounds is a dynamic process that involves chemical reactions, mobility of molecules within drying cells, and sorption/desorption processes. Observed differences in VOC emission among species can be attributed to substrate levels in seeds and varying responses to drying. Different suites of molecules were detected in seeds stored under humid and dry conditions and appear to reflect decreasing propensity for fermentation-type reactions and increasing propensity for triacylglycerol degradation in aqueous and non-aqueous domains, respectively, as cells dry. Our study suggests an indirect association of fermentation-type reactions, but no association of triacylglycerol oxidation, with seed longevity. Longevity was mostly unaffected in mixtures of seeds emitting different VOCs, suggesting that the molecules we detected were not damaging. Our study shows differences among seeds in the adsorption of VOCs on to molecular matrices, mechanisms of fermentation-type reactions under water-stress conditions, and possible effects of moisture on lipid body behavior. Understanding these differences may provide greater insight into the mechanisms by which dry seeds succumb with time.
